# Income inequality and pandemics: insights from HIV/AIDS and COVID-19—a multicountry observational study

**DOI:** 10.1136/bmjgh-2023-013703

**Published:** 2023-09-16

**Authors:** John Ele-Ojo Ataguba, Charles Birungi, Santiago Cunial, Matthew Kavanagh

**Affiliations:** 1 African Health Economics and Policy Association, Accra, Ghana; 2 Health Economics Laboratory, University of Manitoba Faculty of Health Sciences, Winnipeg, Manitoba, Canada; 3 Equitable Financing, Joint United Nations Programme on HIV/AIDS, Nairobi, Kenya; 4 Political Science, University of Pennsylvania, Philadelphia, Pennsylvania, USA; 5 School of Health, Georgetown University, Washington, DC, USA; 6 UNAIDS–Georgetown Collaborating Centre on HIV Policy and Inequality, Joint United Nations Programme on HIV/AIDS, Geneve, Switzerland

**Keywords:** COVID-19, Health policies and all other topics, HIV

## Abstract

**Objectives:**

Assess the relationship between income inequality and HIV incidence, AIDS mortality and COVID-19 mortality.

**Design:**

Multicountry observational study.

**Setting:**

217 countries for HIV/AIDS analysis, 151 countries for COVID-19 analysis.

**Participants:**

Used three samples of national-level data: a sample of all countries with available data (global sample), a subsample of African countries (African sample) and a subsample excluding African countries (excluding African sample).

**Main outcome measures:**

HIV incidence rate per 1000 people, AIDS mortality rate per 100 000 people and COVID-19 excess mortality rate per 100 000 people. The Gini index of income inequality was the primary explanatory variable.

**Results:**

A positive and significant relationship exists between the Gini index of income inequality and HIV incidence across all three samples (p<0.01), with the effect of income inequality on HIV incidence being higher in the African sample than in the rest of the world. Also, a statistically positive association exists for all samples between income inequality and the AIDS mortality rate, as higher income inequality increases AIDS mortality (p<0.01). For COVID-19 excess mortality rate, a positive and statistically significant relationship exists with the Gini index for the entire sample and the excluding African sample (p<0.05), but the African sample alone did not deliver significant results (p<0.1).

**Conclusion:**

COVID-19 excess deaths, HIV incidence and AIDS mortality are significantly associated with income inequality globally—more unequal countries have a higher HIV incidence, AIDS mortality and COVID-19 excess deaths than their more equal counterparts. Income inequality undercuts effective pandemic response. There is an urgent need for concerted efforts to tackle income inequality and to build pandemic preparedness and responses that are adapted and responsive to highly unequal societies, prioritising income inequality among other social determinants of health.

**WHAT IS ALREADY KNOWN ON THIS TOPIC**

- Earlier in the AIDS pandemic, a positive relationship between income inequality and HIV prevalence in sub-Saharan Africa was found.
- The relationship was less clear outside African countries.
- Some single-country studies suggest a link between income inequality and HIV transmission among specific groups.
- Studies primarily from higher income contexts, where data are available, show a positive relationship between income inequality and COVID-19 cases or mortality.

**WHAT THIS STUDY ADDS**

- Income inequality is globally associated with less effective pandemic response across at least two recent pandemics.
- More unequal countries show higher HIV incidence, AIDS mortality and COVID-19 excess mortality.
- The study highlights that both COVID-19 *excess* mortality and AIDS mortality are associated with income inequality at a global level for the first time, including low and middle-income countries often considered to have poor-quality data.

**HOW THIS STUDY MIGHT AFFECT RESEARCH, PRACTICE OR POLICY**

- There is an urgent need to craft more effective pandemic preparedness and response strategies for highly unequal contexts.
- This study highlights the need for global policy responses to tackle income inequality, which is significantly associated with adverse pandemic outcomes.
- Research at the regional or global level should consider income inequality as an essential variable in explaining and attenuating adverse pandemic outcomes.

## Introduction

Pandemics constitute a significant public health problem, posing threats to the health and well-being of substantial population segments across countries, especially marginalised populations. Estimates suggest over 300 excess COVID-19 deaths per 100 000 people as of July 2023^1^ for a pandemic that recorded its first mortality in 2020. The UNAIDS data show that AIDS-related deaths have declined by 52% since 2010. Additionally, in 2022, there were 1.3 million new HIV infections, the fewest since the 1980s, with the declines especially strong in regions with the highest HIV burdens.^2^ Although marginalised populations and countries in the Global South are disproportionately affected, the reality that pandemics do not respect national boundaries calls for collective global action, paying attention to countries with weak infrastructure and vulnerable health systems.^3^ They also necessitate significant solidarity, which is still challenging, as found with COVID-19 vaccines with substantial inequalities in vaccination rates, leaving less wealthy countries behind.^4^

Governments and social entities across various sectors need swift and robust responses. Still, these responses often focus heavily on addressing proximate determinants of health, such as individual behaviours. Yet, it is vital to underline the significance of the broader social determinants of health inequalities which influence these behaviours and health outcomes because, as with many pandemics, including the HIV and COVID-19 pandemics, mortality rates in very deprived areas exceeded that in affluent areas.^5–8^ These social determinants comprise broader policy environment and socioeconomic and environmental factors that indirectly or directly impact health by moulding individuals’ living, schooling, working life, and ageing choices and conditions that are actionable with effective responses.^9^ Despite being less immediately apparent compared with proximate determinants, these social determinants exert considerable influence on health outcomes, often shaping the trajectory of pandemics by affecting disease spread and impact within and across communities.^8 10^ Therefore, their inclusion in pandemic responses is crucial to tackling the root causes of health inequalities, as any policy to change health behaviours cannot substantially reduce health inequalities without tackling the underlying causes outside the health sector.^11^

Several pathways, often linked to class or power structure (including economic, political, social and cultural) within and between societies, exist on how income inequality generally affects health, health outcomes and health inequalities.^12–15^ In the context of pandemics, a likely causal pathway from income inequality to higher rates of pandemic disease within and between countries is evident. Since the 1990s, a large and robust literature comprising several hundred studies links income inequality to health outcomes.^16–21^ A systematic review showed that this evidence includes data meeting epidemiological causality criteria.^22^ Knowing that pandemics exacerbate income and economic inequalities,^8^ this paper highlights three relevant causal pathways from the literature linking income inequality and pandemic infectious diseases. First, inequality can be linked to deprivation among a significant portion of the population in areas from nutrition to education, increasing their vulnerability to infection and disease. In Malawi, for example, higher income inequality was linked to HIV mainly through limited individual choice, higher risk sex and violence,^23^ increasing vulnerability among key populations. Second, inequality is linked to social factors limiting effective pandemic response, including lower social cohesion and trust.^22^ Third, inequality is linked to political factors undermining health, which makes it harder to coordinate an effective response to HIV and COVID-19,^24^ and this weakens the solidarity needed to tackle pandemics.

Increased inequality is hypothesised to be linked to worse pandemic health outcomes, with research highlighting the impact of pandemics on widening inequality^8^ and the ways inequality shaped pandemic responses and subjected specific populations to greater risk and lesser protection in certain countries and regions.^25–28^ However, studies showing how income inequality, a critical social determinant of health inequalities, is associated with major recent pandemics on a global scale are lacking. For the HIV pandemic, most of these studies concentrate on sub-Saharan Africa,^29^ while COVID-19 studies leave out lower income countries^30^ because of claims of data quality for COVID-19 deaths. However, reliable modelled data on COVID-19 excess mortality are now available, providing an avenue to extend the analysis to countries that have been previously excluded. Thus, this paper adds to this literature by assessing the relationship between income inequality and health outcomes (HIV/AIDS and COVID-19, the two most devastating recent pandemics) globally and regionally. It seeks to answer the research question of whether income inequality, measured using the Gini index, is significantly associated with HIV incidence or AIDS mortality and COVID-19 excess mortality. It also highlights the need for concerted efforts to address income inequality and its detrimental effects on pandemic outcomes.

## Methods

### Study design and data sources

Data used for analysis include AIDS mortality rate per 100 000 people, HIV incidence per 1000 people, COVID-19 excess deaths per 100 000 people, Gini index of income inequality, current health expenditure per capita in US$, World Bank income categories and the UNAIDS regions. The data sources included the World Bank,^31^ UNAIDS,^2^ the Economist Intelligence Unit^1^ and the World Inequality Database,^32^ as shown in table 1. For HIV/AIDS analysis, time series data covered 2000–2021, while the COVID-19 analysis covered 2020/2021. Overall, there were 217 countries for the HIV/AIDS analysis and 151 countries for the COVID-19 analysis. However, the actual number of observations for each analysis varies and depends on complete data availability. Because of concerns regarding under-reporting COVID-19 deaths in many countries, especially in Africa and Asia, this paper uses excess mortality caused by COVID-19 as modelled and reported through the Economist Intelligence Unit^1^ mainly because of the transparency and public availability of the underlying codes used to generate excess mortality due to COVID-19 in each country. Notably, the Economist Intelligence Unit used data from sources including Karlinsky and Kobak’s^33^ World Mortality Dataset and the Human Mortality Database.^34^

**Table 1:** Descriptive statistics

Variable	Mean	SD	Min	Max	n	Source
AIDS mortality rate per 100 000 people*	44.456	119.167	0.009	1118.747	3784	UNAIDS^2^
HIV incidence per 1000 people*	0.795	2.206	0.001	21.684	3784	UNAIDS^2^
COVID-19 excess deaths per 100 000 people†	73.359	97.365	151.108	655.319	171	The Economist and Solstad^1^
Gini index*	0.573	0.087	0.370	0.781	3434	World Inequality Database^32^
Health expenditure per capita (US$)*	927.486	1649.671	4.000	11 702.000	3542	World Bank^31^

*Period covered is 2000–2021.

†Period covered in 2020/2021. COVID-19 excess deaths refer to an estimate of the deaths that occurred during the COVID-19 pandemic over and above what would be expected in the absence of the pandemic.

max, maximum value; min, minimum value; n, number of observations.

### Statistical analysis

Analytically, this paper assesses the relationship between income inequality (where the Gini index ranged from 0, a case of perfect equality, to 1 for perfect inequality) and HIV incidence, AIDS mortality and COVID-19 excess mortality using the linear regression model.^35^ The general model can be written as follows:

$$H_{it}=\alpha +\beta_{1}IN_{it}+\beta_{2}X_{it}+\varepsilon_{it}$$

where *H* corresponds to the primary health outcomes (ie, HIV incidence per 1000 people, AIDS mortality rate per 100 000 people or COVID-19 excess mortality per 100 000 people) in country *i* in year *t*. *IN_it_* is the Gini index of income inequality and *X* is the vector of control variables.

For the HIV analysis, *H_it_* is replaced with *lnH_it_*_+1_, the natural logarithm, because this was non-negative, where *t*+1 signifies that values of the following year were used because it is hypothesised that current income inequality is associated with future health outcomes. $\beta_{1}$ is the coefficient associated with our primary indicator of income inequality (Gini index) in country i in year t . $\beta_{2}$ is a vector of coeﬀicients of several alternative factors in country i in year t that impact health outcomes: the country’s income level or category, per capita health expenditure and UNAIDS region. Although economists would argue against allowing income to determine people’s access to health services, it is the case that income level, at the country level and between countries, is a critical determinant of health outcomes and differences in health outcomes between countries.^36^ Higher income typically provides better access to resources needed for a healthier lifestyle, including quality food, housing, education and healthcare services. It can also mitigate the impact of stressors that can negatively impact health. By controlling for income levels using the World Bank income categories of countries, we can separate the effect of income (wealth) from that of inequality since they are inter-related but distinct factors influencing health outcomes. Health expenditure per capita directly measures the resources allocated for health in a country at an individual level. It captures aspects related to the availability and quality of health services, which are crucial factors in health outcomes. Health expenditure per capita may be associated with inequality and health outcomes,^37^ so including it as a covariate prevents omitted variable bias and provides a more accurate estimate of the effect of inequality on health.

The HIV/AIDS models included regional and year fixed effects to control the average differences across regions and years in unobservable predictors.^38^ The COVID-19 model contains only regional fixed effects as the complete data included information on COVID-19 deaths between 2020 and 2021 because the latest available data on per capita health expenditure were in 2021,^31^ and many countries did not record significant COVID-19 mortality until mid-2020. Africa is most severely affected by HIV and AIDS,^2 39^ and the reportedly low COVID-19 mortality in Africa was described as a paradox.^40^ So, even though modelled COVID-19 excess mortality data were used^1^ in the case of COVID-19 estimations, to avoid skewed regression estimates, three separate regression models were estimated for each dependent variable—a global sample, an Africa-only sample and a sample excluding Africa.

All analyses were done in Stata V.17,^41^ and the paper follows the Strengthening the Reporting of Observational Studies in Epidemiology cross-sectional reporting guidelines.^42^

### Patient and public involvement

This study analyses secondary data sets and does not directly involve patients or the public. Although patients were not involved, the findings from the paper have been presented to a broader audience from many countries.

## Results

### Basic and descriptive statistics

Over the period covered in the analysis, the descriptive statistics in table 1 show that the average annual per capita current health expenditure was slightly less than $1000. Income inequality measured by the Gini index ranged from 0.37 (in Hungary, the least unequal case) to 0.78 (in Botswana and Namibia, the most unequal case), with an average Gini index estimated at 0.57. The average AIDS mortality rate was 44.46 deaths per 1 000 000 people, while the average HIV incidence rate was about 0.80 per 1000 people. The average COVID-19 excess mortality was estimated at 73.36 per 100 000 people. The negative value for minimum COVID-19 excess mortality (−151.11 in Seychelles) occurs because the death rate during COVID-19 was lower than expected without the pandemic.

### Income inequality and HIV/AIDS outcomes

The regression results in table 2 are for two broad models. The first segment is for the HIV incidence model, while the second is for the AIDS mortality model. The three separate analyses for each model included the global sample, excluding the African countries and only African countries. The results show a positive and significant relationship between income inequality and the natural logarithm of HIV incidence per 1000 people in the following year across all three samples (p<0.01). As predicted, higher levels of income inequality, measured using the Gini index, are significantly associated with an increased incidence rate of HIV per 1000 people in the following year (p<0.01). Loosely speaking, overall and in Africa, higher levels of income inequality in a year are associated with higher HIV incidence in the next year, all things being equal. Specifically, the effect of income inequality on HIV incidence was higher in the African subsample than in the rest of the world, probably due to higher HIV rates in the sub-Saharan Africa region.

**Table 2:** Income inequality and HIV/AIDS outcomes

	HIV incidence model			AIDS mortality model		
	Global sample	Excluding Africa	Only Africa	Global sample	Excluding Africa	Only Africa
Gini index	6.31*** (0.384)	4.70*** (0.464)	8.48*** (0.715)	7.62*** (0.462)	8.33*** (0.604)	6.13*** (0.759)
Covariates	Yes	Yes	Yes	Yes	Yes	Yes
UNAIDS regions and year fixed effects	Yes	Yes	Yes	Yes	Yes	Yes
Observations (n)	3183	2211	972	3283	2211	972
HIV incidence (t+1) and AIDS mortality (t+1) response to 25% reduction in Gini index	0.14*** [0.007]	0.29*** [0.005]	2.11*** [0.048]	6.58*** [0.341]	17.39*** [0.174]	11.45*** [3.191]

Dependent variables were the natural logarithm of HIV incidence rate per 1000 people at time t+1 and the natural logarithm of AIDS mortality rate per 100 000 at time t+1.

Analytical SEs in parenthesis (); bootstrapped SEs using 500 replications in square brackets []; ***p<0.01.

Covariates include current health expenditure per capita and World Bank income categories (low, lower middle, upper middle and upper income countries); UNAIDS regions included East and Southern Africa, West and Central Africa, Asia and Pacific, Eastern Europe and Central Asia, Latin America and the Caribbean, North Africa and the Middle East, West and Central Europe and North America.

Applying Duan’s smearing estimator^43^ to the results in table 2, a 25 percentage point reduction in the Gini index corresponds to the HIV incidence rate for the next year, significantly dropping by 0.14 per 1000 people for the global sample (p<0.01). For the African subsample, this will significantly reduce the HIV incidence rate by 2.11 per 1000 people in the next year (p<0.01). Similarly, for AIDS mortality, a 25% reduction in the Gini index is associated with a significant decline in AIDS mortality rate by 6.58 (p<0.01), 11.45 (p<0.01) and 17.39 (p<0.01) per 100 000 people in the next year for the global sample, the African sample and the sample excluding Africa, respectively.

**Figure 1:**
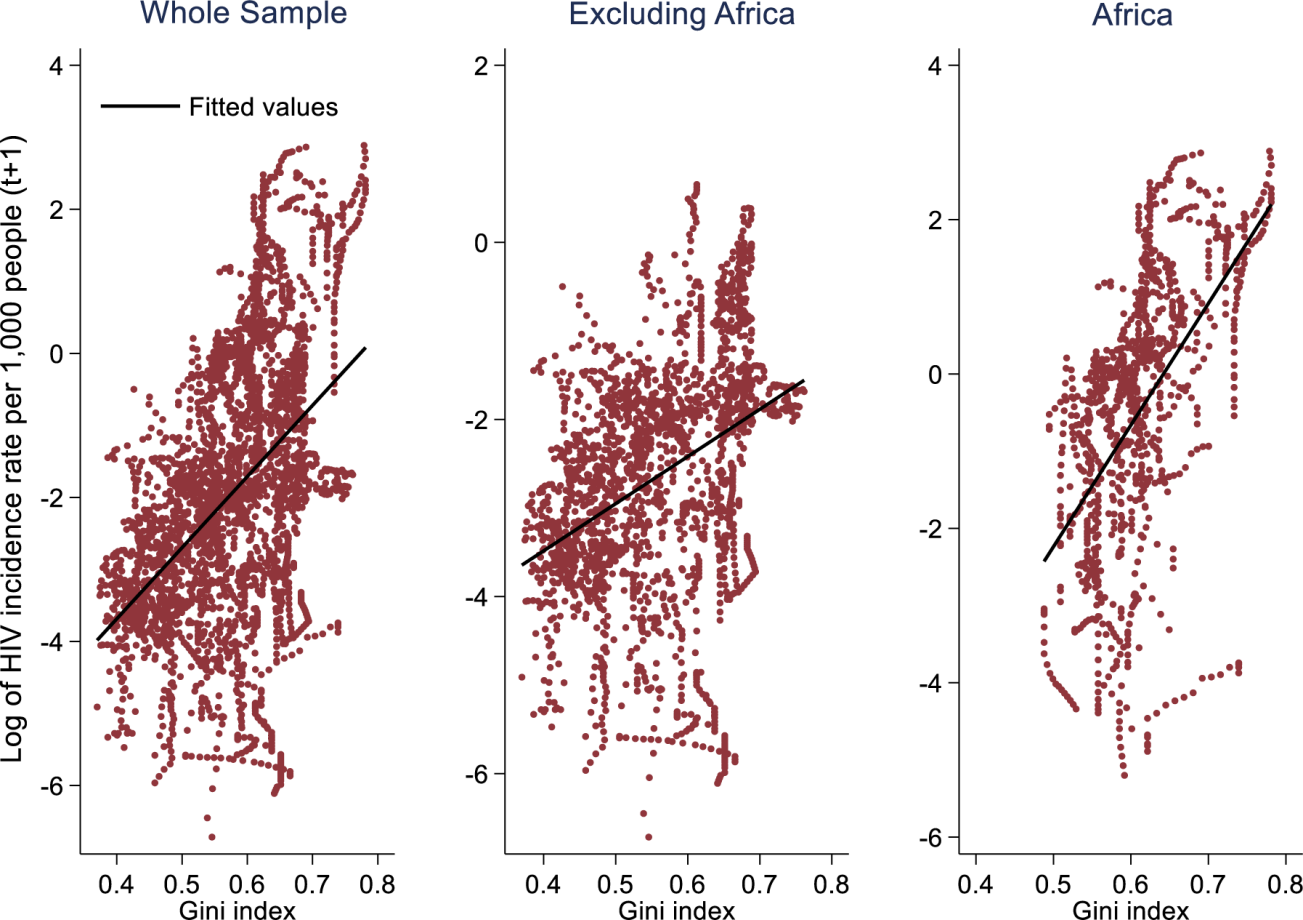
Income inequality and HIV incidence, 2020–2021.

Figure 1 shows the positive relationship between income inequality and the natural logarithm of HIV incidence per 1000 people, with a steeper slope for the African subsample.

**Figure 2:**
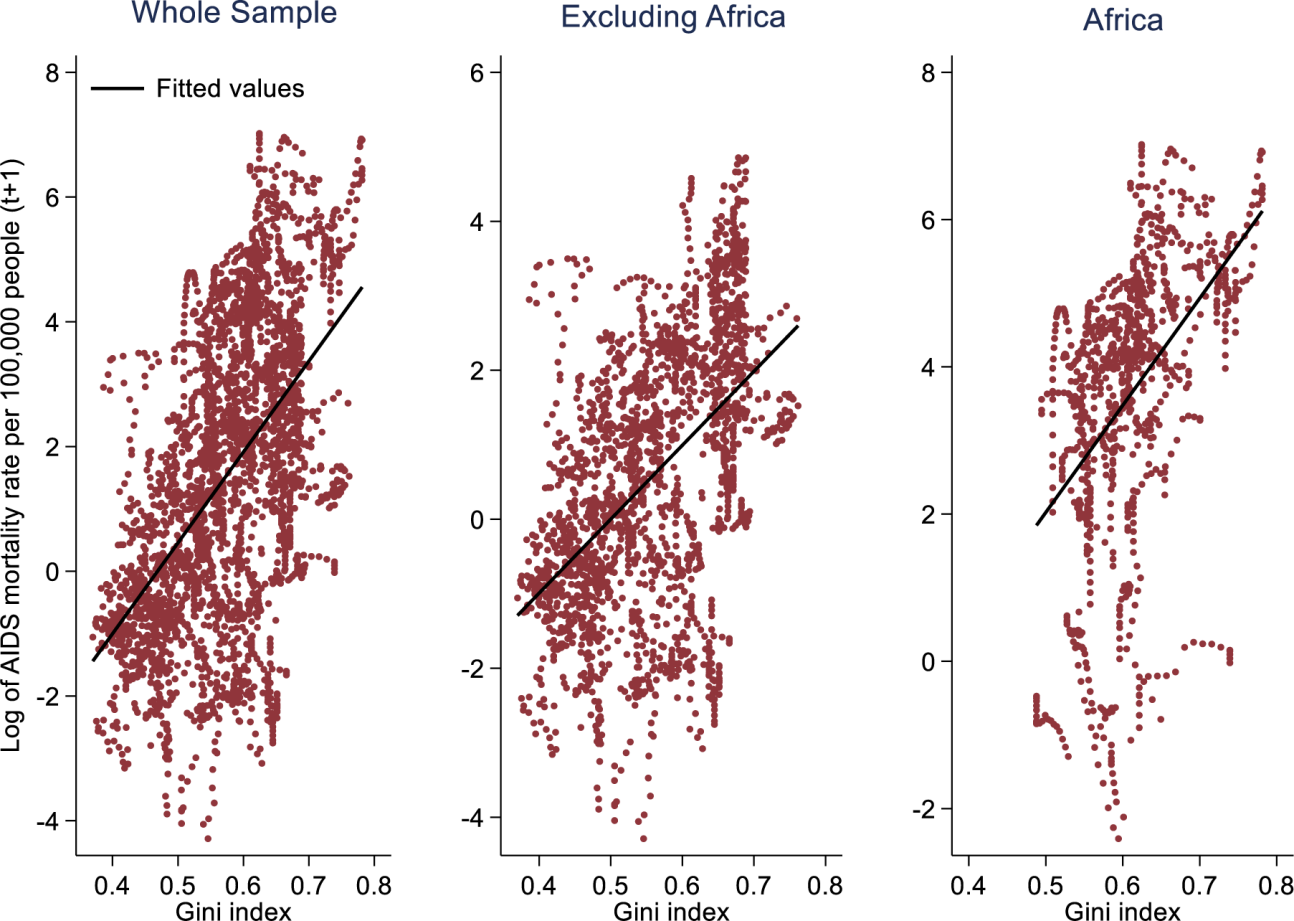
Income inequality and AIDS mortality, 2000–2021.

Similar patterns emerge when examining the relationship between income inequality and the AIDS mortality rate per 100 000 people. A positive association between income inequality, as measured by the Gini index, and the natural logarithm of AIDS deaths per 100 000 people (figure 2) exists as higher levels of income inequality in a year are linked to an increase in next year’s AIDS mortality rate (p<0.01). Unlike the results for HIV incidence, the effect size was not highest in the African subsample even though it is statistically significant at the 1% significance level. Reducing income inequality (ie, the Gini index by 25%) is significantly associated with a reduction in the AIDS mortality rate by 6.58 (p<0.01) and 11.45 (p<0.01) deaths per 100 000 people in the following year for the entire sample and the African subsample, respectively.

### Income inequality and COVID-19

**Figure 3:**
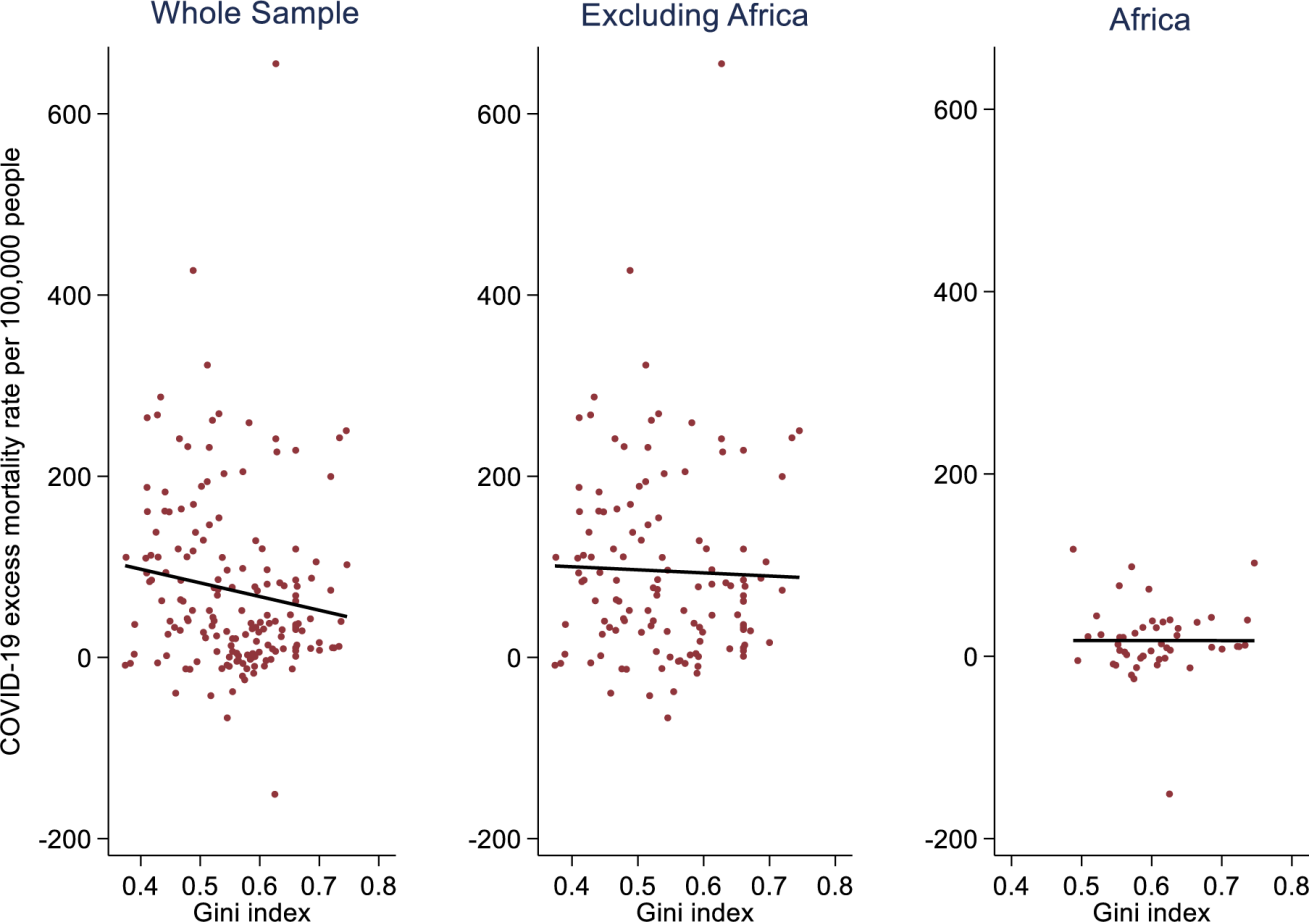
Income inequality and COVID-19 excess mortality, 2020/2021.

The relationship between income inequality (using the Gini index) and COVID-19 excess mortality rate per 100 000 people, as shown in figure 3, was not initially positive for the entire sample. However, a positive relationship emerged using the regression model presented in table 3.

**Table 3:** Income inequality and COVID-19 excess mortality

	Global sample	Excluding Africa	Only Africa
Gini index	246.30** (110.30)	331.10** (154.00)	80.71 (63.14)
Covariates	Yes	Yes	Yes
UNAIDS regions fixed effects	Yes	Yes	Yes
Observations (n)	171	123	48
COVID-19 excess mortality response to 25% reduction in Gini index	−35.27 [23.11]	−11.55 [12.02]	−47.43 [33.55]

Dependent variable is COVID-19 excess mortality rate per 100 000 people.

Analytical SEs in parenthesis (); bootstrapped SEs using 500 replications in square brackets []; **p<0.05.

Covariates include current health expenditure per capita and World Bank income categories (low, lower middle, upper middle and upper income countries); UNAIDS regions included East and Southern Africa, West and Central Africa, Asia and Pacific, Eastern Europe and Central Asia, Latin America and the Caribbean, North Africa and the Middle East, West and Central Europe and North America.

After controlling health expenditure per capita, regions and income groups, a positive and statistically significant relationship between the Gini index and COVID-19 excess mortality rate per 100 000 people was found for the entire sample and the subsample excluding Africa (p<0.05), as shown in table 3. This result means that more unequal countries tend to report more COVID-19 excess mortality than their more equal counterparts, all other things being equal. The results for the African subsample were not statistically significant (p>0.1), even though a positive relationship was found. This may be partly due to a smaller sample of countries^44^ and the near homogeneity in the distribution of COVID-19 excess mortality for the African subsample in figure 3. A sensitivity analysis was conducted for Africa’s subsample by excluding Seychelles’ data point with −151.1 COVID-19 excess mortality, and the results remain remarkably similar, still not statistically significant.

## Discussion

This paper highlights the significant relationship between income inequality measured using the Gini index and key pandemic variables, including HIV incidence, AIDS mortality and COVID-19 excess mortality. It emerged that unequal societies are burdened more by HIV/AIDS and COVID-19 excess deaths than their more equal counterparts, with the results remaining similar for Africa and the rest of the world. These findings show that income inequality is significantly and adversely associated with pandemic outcomes. The robust association between higher levels of inequality and critical indicators such as HIV incidence per 1000 people, AIDS mortality per 100 000 people and COVID-19 excess mortality suggests income inequality is a factor that may limit effective pandemic response because income inequality creates conditions that perpetuate and exacerbate pandemics, leaving marginalised populations more vulnerable to the devastating consequences of pandemics.

### Strengths and weaknesses of this study

A major strength of the analyses in this paper is its multicountry nature, with data from all the UNAIDS regions with available data. Also, the paper uses a standardised measure of income inequality, the Gini index, to assess the relationship between income inequality and pandemic health outcomes. The paper was not limited to one pandemic outcome but considered three outcomes. As a limitation, this paper focused solely on income inequality and its relationship with pandemic outcomes. It did not delve into other essential dimensions of inequality, such as disparities based on gender, race, sexuality and other key population characteristics, which often intersect with income inequality, exacerbating the challenges of marginalised communities and increasing the burden of health disparities.^45 46^ Another limitation of the paper is that it did not assess causality, as the results imply association. But the significance of the relationships in many cases signifies the strength of the association between income inequality and pandemic health outcomes. In turn, the results represent an initial step for future investigations that delve into the robustness of the relationship between inequality and pandemic outcomes.

Although this study was about global phenomena like pandemics, requiring comparable information and data from diverse countries and societies, data availability and quality posed a significant challenge. It is important to note that this analysis cannot discount how disparities in data collection methods, reporting accuracy, and scope across different regions and periods could impact the reliability and comparability of results. For example, the COVID-19 analysis relied on excess mortality data based on epidemiological modelling because of the paucity of COVID-19 mortality data in Africa and Asia, where reported COVID-19 mortality was scanty and not comprehensive. While the HIV/AIDS analyses were based on time series data, the COVID-19 analysis uses almost cross-sectional data, limiting the analysis power. It is undisputed that numerous factors (at the microlevel, mesolevel and macrolevel) other than those controlled in this paper’s analyses influence pandemic outcomes. For example, health system capacities, country-level policy responses and heterogeneities, governance quality, cultural behaviours and pre-existing health conditions play substantial roles in determining pandemic responses and outcomes. These variables may interact with income inequality in complex ways, adding complexity to the analysis. These notwithstanding, this paper serves as a starting point to take this research forward to understand how complex factors interact and the pathways through which income inequalities affect pandemic outcomes.

### Comparison with other studies

This study breaks new ground on several fronts—presenting truly global associations between income inequality and multiple pandemic outcomes, using updated AIDS mortality and COVID-19 excess mortality data to enable wide coverage of low and middle-income countries, and doing so with data accounting for major shifts in recent decades in the AIDS response.

Most studies on HIV from earlier in the pandemic’s history did not focus on inequality per se, but rather on poverty and wealth. This focus was particularly driven by surprising evidence from some population surveys suggesting that HIV infection was not consistently higher among the poor; in fact, it was often more prevalent among wealthier individuals^44 47 48^ and is higher in Africa’s wealthiest countries.^49^ Some smaller studies using data from sub-Saharan Africa^29 50–52^ show a significant relationship between income inequality and HIV prevalence, and occasionally transmission. Income inequality, for example, has been linked to the spread of HIV among women in Malawi at the neighbourhood and district levels^23^ and persons who inject drugs in Vietnam at the community level.^53^ In a smaller area setting, New York City in the USA, the relationship between income inequality and adverse HIV health outcomes has been reported.^45^ In contrast, a study in Asia (one of the only analyses outside Africa) found no association between income and HIV prevalence.^48^ Most use HIV prevalence, a less meaningful measure we would argue than HIV incidence. Meanwhile, there is a paucity of literature on the income inequality and AIDS mortality nexus. There is evidence of higher risk of mortality among persons of low socioeconomic status in South Africa and the USA,^54 55^ but to the best of our knowledge, there is no literature that discusses the relationship between income inequality and AIDS mortality, particularly at the global level. In addition, studies have not considered the most recent decade of data, during a time a significant change in the AIDS pandemic. Our paper uses the most recent data to bridge these gaps. This paper demonstrates a statistically significant relationship between inequality and both new HIV infections and AIDS deaths through to the present time and does so beyond a country or region to present evidence at a global level.

While the COVID-19 pandemic could exacerbate income and economic inequality,^56^ a significant positive relationship between income inequality and COVID-19 cases or mortality has also been reported.^30 46 57 58^ Most studies have focused on a smaller subset of countries including Organisation for Economic Co-operation and Development member countries where data are robust. These findings largely align those reported in this paper for COVID-19 excess mortality. Notably, there is a dearth of studies using COVID-19 excess mortality, considered to reflect better the impact of COVID-19 deaths over and above the expected death rates within countries.^59^ In particular, many low and middle-income countries have been excluded from previous studies assessing the association between COVID-19 health outcomes and income inequality mainly because of data paucity. Although the relationship between income inequality and COVID-19 excess mortality was not significant for Africa, the overall similarity of this paper’s findings regarding the role of income inequality in aggravating the effects of the COVID-19 pandemic is critical to note.

### Implications of findings

The correlation between income inequality and other social variables demonstrates the ways that inequality undermines pandemic response. More unequal countries are likely to have higher HIV and COVID-19 rates both because of unequal access to essential health services and other relevant services outside the health sector, including education and shelter.^23^ Beyond reporting the association between income inequality and pandemic outcomes, which is crucial, it is imperative to understand the mechanisms more deeply through which income inequality influences health outcomes amidst a pandemic. For the AIDS pandemic, the means of influence likely operate on multiple levels. At a social and political level, widening income inequality may foster greater *othering* in pandemic response both within and between countries, which undermines universal responses and the deprioritisation of services, disproportionately serving a disfavoured portion of the population, as has been shown in some contexts.^60^ As those with good healthcare access, living in well-off communities, get pre-exposure prophylaxis through private means, for instance, HIV rates for such communities may fall rapidly. At the same time, the political priority for reaching the rest diminishes. At an individual level, income levels and inequality may reduce choices for preventing HIV transmission—for example, by pushing more people into higher HIV risk or transactional sex. In other words, the relationship between income inequality and pandemics means that despite the widespread availability of HIV testing, prevention and treatment options, societies with greater inequality have struggled to use these resources to reduce rates of HIV infection and AIDS-related deaths effectively.

Meanwhile, the overall health of people, which has been concretely tied to various social determinants,^23^ likely makes people in unequal societies more vulnerable to HIV mortality and less able to access good HIV care and cutting edge medicines.^61^ Similar mechanisms likely apply to the COVID-19 pandemic. The COVID-19 pandemic presented a unique challenge as it led to many drastic measures undertaken by governments. Wealthier countries with more resources could provide relief and better policy responses than their less affluent counterparts.^3^ The relationship between COVID-19 excess mortality and income inequality likely means that more unequal countries were challenged to ensure access to preventive measures, testing and treatment, including vaccination,^62^ leading to elevated COVID-19 excess mortality rates in these countries relative to less unequal countries. In the subsample of African countries, the insignificant relationship between income inequality and COVID-19 excess deaths may not be surprising due to the smaller number of observations and relatively low variability in the Gini index of income inequality compared with the subsample that excludes African countries. This further supports inequality’s role in exacerbating COVID-19 excess mortality, especially in a sample of unequal countries. Indeed, the data seem to suggest that economic inequality is a more significant driving force than net wealth.

Based on the findings of this study, to effectively respond to future health crises, it is crucial to prioritise policies and interventions that aim to reduce income inequality. But pandemics will not wait for changes in economic distribution. As such, it is crucial to consider how pandemic responses in highly unequal societies could act to counter, rather than reinforce, those inequalities. Although the results from this study show that income inequality is bad for effective pandemic responses using HIV/AIDS and COVID-19 pandemics, as noted in the limitations of this paper, focusing on income inequalities is just one aspect of broader social inequality. Future research should incorporate additional dimensions of inequality to fully understand the complex dynamics of pandemics and their societal impacts. This comprehensive approach will help develop more effective and equitable pandemic responses, promote social justice and improve health outcomes across diverse segments of society. By taking a holistic approach, we can build a more resilient and equitable society better equipped to mitigate the devastating effects of future pandemics.

## Conclusion

Income inequality significantly correlates with HIV incidence, AIDS mortality rate and COVID-19 excess mortality rate using a cross-country analysis involving the UNAIDS regions. The findings reported in this paper underscore the urgent need for concerted efforts to tackle income inequality and its detrimental effects on pandemic outcomes and to craft pandemic responses that work more effectively in highly unequal contexts—countering rather than reinforcing inequality. We argue that this sits at the heart of the global call to leave no population behind in attaining development goals.

## Data Availability

Data are available in a public, open access repository. All data used in this paper are available in the public domain from different sources. HIV/AIDS data are available from the UNAIDS (https://aidsinfo.unaids.org/), COVID-19 data from The Economist (https://www.economist.com/graphic-detail/coronavirus-excess-deaths-estimates), health expenditure data from the World Bank (https://data.worldbank.org/indicator) or WHO, and the Gini index of income inequality from the World Inequality Database (https://wid.world/data/).

## References

[1] Solstad SU , The Economist . Data from: the pandemic’s true death toll: COVID-19 data. data repository [The Economist]. n.d. Available: https://www.economist.com/graphic-detail/coronavirus-excess-deaths-estimates

[2] UNAIDS . Aidsinfo: UNAIDS. 2023. Available: https://aidsinfo.unaids.org/

[3] Ataguba JE . COVID-19 pandemic, a war to be won: understanding its economic implications for Africa. Appl Health Econ Health Policy 2020;18:325–8. 10.1007/s40258-020-00580-x 32249362 PMC7130452

[4] Hunter DJ , Abdool Karim SS , Baden LR , et al . Addressing vaccine inequity—COVID-19 vaccines as a global public good. N Engl J Med 2022;386:1176–9. 10.1056/NEJMe2202547 35196425

[5] McGowan VJ , Bambra C . COVID-19 mortality and deprivation: pandemic, syndemic, and endemic health inequalities. Lancet Public Health 2022;7:e966–75. 10.1016/S2468-2667(22)00223-7 36334610 PMC9629845

[6] Piot P , Bartos M , Ghys PD , et al . The global impact of HIV/AIDS. Nature 2001;410:968–73. 10.1038/35073639 11309626

[7] Deaton A . Policy implications of the gradient of health and wealth. Health Affairs 2002;21:13–30. 10.1377/hlthaff.21.2.13 11900153

[8] Paremoer L , Nandi S , Serag H , et al . Covid-19 pandemic and the social determinants of health. BMJ 2021;372:n129. 10.1136/bmj.n129 33509801 PMC7842257

[9] Marmot M . Social determinants of health inequalities. Lancet 2005;365:1099–104. 10.1016/S0140-6736(05)71146-6 15781105

[10] Ataguba OA , Ataguba JE . Social determinants of health: the role of effective communication in the COVID-19 pandemic in developing countries. Glob Health Action 2020;13:1788263. 10.1080/16549716.2020.1788263 32657669 PMC7480618

[11] McCartney G , Collins C , Mackenzie M . What (or who) causes health inequalities: theories, evidence and implications? Health Policy 2013;113:221–7. 10.1016/j.healthpol.2013.05.021 23810172

[12] Coburn D . Income inequality, social cohesion and the health status of populations: the role of neo-liberalism. Soc Sci Med 2000;51:135–46. 10.1016/s0277-9536(99)00445-1 10817476

[13] Coburn D . Beyond the income inequality hypothesis: class, neo-liberalism, and health inequalities. Soc Sci Med 2004;58:41–56. 10.1016/s0277-9536(03)00159-x 14572920

[14] Kawachi I , Kennedy BP . Health and social cohesion: why care about income inequality. BMJ 1997;314:1037–40. 10.1136/bmj.314.7086.1037 9112854 PMC2126438

[15] Kawachi I , Kennedy BP , Lochner K , et al . Social capital, income inequality, and mortality. Am J Public Health 1997;87:1491–8. 10.2105/ajph.87.9.1491 9314802 PMC1380975

[16] Deaton A . Health, inequality, and economic development. J Econ Lit 2003;41:113–58. 10.1257/jel.41.1.113

[17] Subramanian SV , Kawachi I . Income inequality and health: what have we learned so far Epidemiol Rev 2004;26:78–91. 10.1093/epirev/mxh003 15234949

[18] Wilkinson RG , Pickett KE . Income inequality and population health: a review and explanation of the evidence. Soc Sci Med 2006;62:1768–84. 10.1016/j.socscimed.2005.08.036 16226363

[19] Wilkinson RG , Pickett KE . The problems of relative deprivation: why some societies do better than others. Soc Sci Med 2007;65:1965–78. 10.1016/j.socscimed.2007.05.041 17618718

[20] Wilkinson RG , Pickett KE . Income inequality and socioeconomic gradients in mortality. Am J Public Health 2008;98:699–704. 10.2105/AJPH.2007.109637 17901426 PMC2376999

[21] Macinko JA , Shi L , Starfield B , et al . Income inequality and health: a critical review of the literature. Med Care Res Rev 2003;60:407–52. 10.1177/1077558703257169 14677219

[22] Pickett KE , Wilkinson RG . Income inequality and health: a causal review. Soc Sci Med 2015;128:316–26. 10.1016/j.socscimed.2014.12.031 25577953

[23] Durevall D , Lindskog A . Economic inequality and HIV in Malawi. World Development 2012;40:1435–51. 10.1016/j.worlddev.2011.12.003

[24] Lynch J . Regimes of inequality: the political economy of health and wealth. Cambridge: Cambridge University Press, 2019. 10.1017/9781139051576

[25] Nwosu CO , Oyenubi A . Income-related health inequalities associated with the coronavirus pandemic in South Africa: a decomposition analysis. Int J Equity Health 2021;20:21.:21. 10.1186/s12939-020-01361-7 33413442 PMC7790046

[26] Bambra C , Lynch J , Smith KE . The unequal pandemic: COVID-19 and health inequalities. Bristol: Policy Press, 2021. 10.46692/9781447361251

[27] Ohlbrecht H , Jellen J . Unequal tensions: the effects of the Coronavirus pandemic in light of subjective health and social inequality dimensions in Germany. European Societies 2021;23:S905–22. 10.1080/14616696.2020.1852440

[28] Ali S , Asaria M , Stranges S . COVID-19 and inequality: are we all in this together Can J Public Health 2020;111:415–6. 10.17269/s41997-020-00351-0 32578185 PMC7310590

[29] Tsafack Temah C . What drives HIV/AIDS epidemic in sub-Saharan Africa Revue D’économie Du Développement? 2010;17:41–70. 10.3917/edd.235.0041

[30] Elgar FJ , Stefaniak A , Wohl MJA . The trouble with trust: time-series analysis of social capital, income inequality, and COVID-19 deaths in 84 countries. Soc Sci Med 2020;263:113365. 10.1016/j.socscimed.2020.113365 32981770 PMC7492158

[31] World Bank . Digital repository: world data bank. Washington D.C: World Bank; 2023. Available: https://data.worldbank.org/indicator/SH.XPD.CHEX.PC.CD

[32] Chancel L , Piketty T , Saez E , et al . Data from: World Inequality Report 2022. World Inequality Database. 2022. 10.4159/9780674276598

[33] Karlinsky A , Kobak D . Tracking excess mortality across countries during the COVID-19 pandemic with the world mortality dataset. Elife 2021;10:e69336. 10.7554/eLife.69336 34190045 PMC8331176

[34] Human Mortality Database . Human mortality database: reliability and accuracy matter. 2023. Available: https://www.mortality.org

[35] Wooldridge JM . Econometric analysis of cross section and panel data. London: The MIT press, 2010.

[36] Cookson R , Doran T , Asaria M , et al . The inverse care law re-examined: a global perspective. Lancet 2021;397:828–38. 10.1016/S0140-6736(21)00243-9 33640069

[37] Dieleman JL , Sadat N , Chang AY , et al . Trends in future health financing and coverage: future health spending and universal health coverage in 188 countries, 2016–40. Lancet 2018;391:1783–98. 10.1016/S0140-6736(18)30697-4 29678341 PMC5946843

[38] Allison PD . Fixed effects regression models. California: SAGE publications, 2009. 10.4135/9781412993869

[39] IHME . GBD compare | Viz Hub Washington: Institute for health Metrics and evaluation (IHME). 2020. Available: https://vizhub.healthdata.org/gbd-compare

[40] Lawal Y . Africa's low COVID-19 mortality rate: a paradox? Int J Infect Dis 2021;102:118–22. 10.1016/j.ijid.2020.10.038 33075535 PMC7566670

[41] StataCorp . Stata: release 17 - Statistical software. College Station, Texas: StataCorp LP, 2021.

[42] Elm E von , Altman DG , Egger M , et al . The strengthening the reporting of observational studies in epidemiology (STROBE) statement: guidelines for reporting observational studies. BMJ 2007;335:806–8. 10.1136/bmj.39335.541782.AD 17947786 PMC2034723

[43] Duan N . Smearing estimate: a nonparametric retransformation method. JASA 1983;78:605–10. 10.1080/01621459.1983.10478017

[44] Shelton JD , Cassell MM , Adetunji J . Is poverty or wealth at the root of HIV? Lancet 2005;366:1057–8. 10.1016/S0140-6736(05)67401-6 16182881

[45] Ransome Y , Kawachi I , Braunstein S , et al . Structural inequalities drive late HIV diagnosis: the role of black racial concentration, income inequality, socioeconomic deprivation, and HIV testing. Health & Place 2016;42:148–58. 10.1016/j.healthplace.2016.09.004 27770671 PMC5584790

[46] Su D , Alshehri K , Pagán J . Income inequality and the disease burden of COVID-19: survival analysis of data from 74 countries. Prev Med Rep 2022;27:101828. 10.1016/j.pmedr.2022.101828 35581989 PMC9101697

[47] Mishra V , Assche SB-V , Greener R , et al . HIV infection does not disproportionately affect the poorer in sub-Saharan Africa. AIDS 2007;21 Suppl 7:S17–28. 10.1097/01.aids.0000300532.51860.2a 18040161

[48] Greener R , Sarkar S . Risk and vulnerability: do socioeconomic factors influence the risk of acquiring HIV in Asia? AIDS 2010;24 Suppl 3:S3–11. 10.1097/01.aids.0000390084.37812.30 20926925

[49] Fox AM . The social determinants of HIV serostatus in sub-Saharan Africa: an inverse relationship between poverty and HIV? Public Health Rep 2010;125 Suppl 4:16–24. 10.1177/00333549101250S405 PMC2882971 20629252

[50] Deuchert E , Brody S . Lack of autodisable syringe use and health care indicators are associated with high HIV prevalence: an international ecologic analysis. Ann Epidemiol 2007;17:199–207. 10.1016/j.annepidem.2006.09.005 17174567

[51] Gillespie S , Kadiyala S , Greener R . Is poverty or wealth driving HIV transmission AIDS 2007;21 Suppl 7:S5–16. 10.1097/01.aids.0000300531.74730.72 18040165

[52] Piot P , Greener R , Russell S . Squaring the circle: AIDS, poverty, and human development. PLoS Med 2007;4:1571–5. 10.1371/journal.pmed.0040314 17958469 PMC2039763

[53] Lim TW , Frangakis C , Latkin C , et al . Community-level income inequality and HIV prevalence among persons who inject drugs in Thai Nguyen, Vietnam. PLoS One 2014;9:e90723. 10.1371/journal.pone.0090723 24618892 PMC3949692

[54] Probst C , Parry CDH , Rehm J . Socio‐economic differences in HIV/AIDS mortality in South Africa. Trop Med Int Health 2016;21:846–55. 10.1111/tmi.12712 27118253

[55] Rubin MS , Colen CG , Link BG . Examination of inequalities in HIV/AIDS mortality in the United States from a fundamental cause perspective. Am J Public Health 2010;100:1053–9. 10.2105/AJPH.2009.170241 20403885 PMC2866621

[56] Angelov N , Waldenström D . COVID-19 and income inequality: evidence from monthly population registers. J Econ Inequal 2023;2023:1–29. 10.1007/s10888-022-09560-8 PMC10015130 37360569

[57] Wildman J . COVID-19 and income inequality in OECD countries. Eur J Health Econ 2021;22:455–62. 10.1007/s10198-021-01266-4 33590424 PMC7883879

[58] Oronce CIA , Scannell CA , Kawachi I , et al . Association between state-level income inequality and COVID-19 cases and mortality in the USA. J Gen Intern Med 2020;35:2791–3. 10.1007/s11606-020-05971-3 32583336 PMC7313247

[59] Beaney T , Clarke JM , Jain V , et al . Excess mortality: the gold standard in measuring the impact of COVID-19 worldwide J R Soc Med 2020;113:329–34. 10.1177/0141076820956802 32910871 PMC7488823

[60] Lieberman ES . Boundaries of contagion: How ethnic politics have shaped government responses to AIDS. Princeton: Princeton University Press, 2009. 10.1515/9781400830459

[61] Haacker M , Birungi C . Poverty as a barrier to antiretroviral therapy access for people living with HIV/AIDS in Kenya. Afr J AIDS Res 2018;17:145–52. 10.2989/16085906.2018.1475401 30003850

[62] Tatar M , Shoorekchali JM , Faraji MR , et al . COVID-19 vaccine inequality: a global perspective. J Glob Health 2022;12:03072. 10.7189/jogh.12.03072 36227706 PMC9559176

